# Serum IGFBP-2 in systemic sclerosis as a prognostic factor of lung dysfunction

**DOI:** 10.1038/s41598-021-90333-0

**Published:** 2021-05-25

**Authors:** Julien Guiot, Makon-Sébastien Njock, Béatrice André, Fanny Gester, Monique Henket, Dominique de Seny, Catherine Moermans, Michel G. Malaise, Renaud Louis

**Affiliations:** 1 Laboratory of Pneumology, GIGA Research Center, University of Liège, University Hospital of Liège, Liège, Belgium; 2 Laboratory of Rheumatology, GIGA Research Center, University of Liège, University Hospital of Liège, Liège, Belgium

**Keywords:** Prognostic markers, Systemic sclerosis, Respiratory tract diseases

## Abstract

Systemic sclerosis (SSc) is a rare connective tissue disease associated with rapid evolving interstitial lung disease (ILD), driving its mortality. Specific biomarkers associated with the progression of this lung disease are highly needed. We aimed to identify specific biomarkers of SSc-ILD to predict the evolution of the disease. For this, we compared prospectively serum levels of several biomarkers associated with lung fibrosis in SSc patients (n = 102), among which SSc-no ILD (n = 63) and SSc-ILD (n = 39), compared to healthy subjects (HS) (n = 39). We also performed a longitudinal study in a subgroup of 28 patients analyzing biomarkers variations and pulmonary function tests over a period of 2 years. Serum level of IGFBP-2 was significantly increased in SSc patients compared to HS, and negatively correlated with pulmonary function (assessed by carbon monoxide transfer coefficient (KCO)) (r = − 0.29, p < 0.01). Two-year longitudinal analysis in a subgroup of 28 SSc patients determined that IGFBP-2 variation was positively correlated with KCO at 2-year follow-up (r = 0.6, p < 0.001). SSc patients with a lower variation of IGFBP-2 (less than 22%) presented significant deterioration of pulmonary function at 2-year follow-up (p < 0.01). ROC curve analysis enabled us to identify that baseline IGFBP-2 > 105 ng/ml was associated with a poor outcome (KCO < 70% predicted) at 2-year follow-up (AUC = 0.75, p < 0.05). We showed for the first time that serum levels of IGFBP-2 might be a prognostic factor of the development of SSc-ILD.

## Introduction

Systemic sclerosis (SSc) is a complex systemic disease of unknown origin associated with a multi-organic affection involving a complex interplay of microvasculopathy, disturbances in fibroblastic function and abnormalities of the immune system^1–3^. While any organ may be involved in the disease process, pulmonary complications of SSc, including interstitial lung disease (ILD) and pulmonary hypertension (PH), remain one of the major causes of morbidity and mortality in the disease^4–7^. Indeed, ILD and PH represent together 60% of SSc-related deaths^8^. SSc-ILDs have many common clinical and pathological characteristics with some other major ILDs, mainly idiopathic pulmonary fibrosis (IPF)^9–12^. Lung fibrosis is present in approximately 25% of SSc patients^13^. Contrary to what is seen in IPF^14^, treatment is mainly based on an aggressive immunosuppressive therapy specifically proposed in the progressive forms of SSc-ILD^15–17^. One of the major problem clinicians have to deal with is to identify patients with increased risk of ILD progression for early intervention^6,18–21^. In this context, prognostic biomarkers are highly needed in order to help clinicians to predict ILD development and provide adequate treatment.

To date, the most frequently used diagnostic biomarkers for SSc are serum autoantibodies. Indeed, more than 90% of SSc patients harbor antinuclear antibodies (ANA) in their serum^22–24^. Some of these are highly specific for SSc, including anti-Scl-70 (also called anti-topoisomerase I) and anti-centromere (anti-CENP-B) antibodies^25,26^. Although ANA are historical biomarkers available for SSc, they are not able to predict the occurrence of ILD. Several serum biomarkers, including surfactant protein-D (SP-D)^27,28^, Krebs Von Den Lungen 6 (KL-6)^29,30^ and chemokine ligand-18 (CCL18), have been associated with SSc-ILD. Furthermore, transforming growth factor beta (TGF-β) is known to be involved in the pathophysiology of many lung fibrotic diseases by stimulating the deposition of collagen and increasing lung remodeling^31,32^. Besides TGF-β, previous studies identified that insulin-like growth factor-binding proteins (IGFBPs) were also clearly associated with IPF and of interest as new potential biomarkers for SSc-ILD^33,34^. IGFBPs are a group of secreted proteins which serve as transport proteins for insulin-like growth factors (IGFs) with high affinity, regulating the bioavailability and function of IGFs^35–38^. IGFBP-2 was found to be increased in the bronchoalveolar lavage (BAL) of children with ILD^39^ and in serum and sputum of IPF patients^20,40^.

The aim of our study was to quantify serum level of several SSc- and IPF-associated growth factors in SSc patients in order to identify novel biomarkers to predict the occurrence of ILD.

## Results

### Study population, patient characteristics, and clinical data

**Figure 1:**
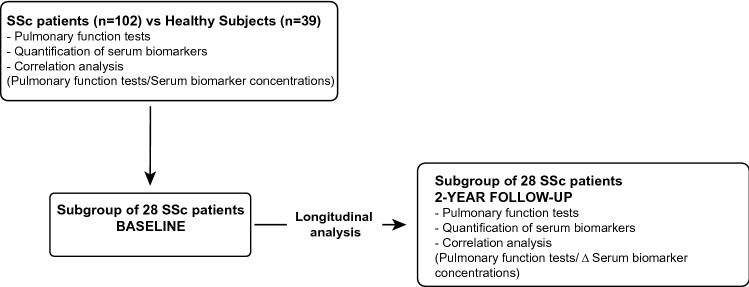
Study design.

We prospectively recruited patients with SSc (SSc-no ILD, n = 63; SSc-ILD, n = 39) from our ambulatory care policlinic at CHU Liege and compared them to healthy subjects (HS) (n = 39) (Fig. 1). Demographic, functional and treatment characteristics of the subjects are given in Table 1. The average age of patients compared to HS was similar. Forced expired volume in 1 s (FEV1) was moderately lowered in the SSc-no ILD and SSc-ILD patients compared to HS (p < 0.05 and p < 0.05, respectively). SSc-ILD patients present lower levels of FEV1, forced vital capacity (FVC), total lung capacity (TLC) and diffusion lung capacity for CO (DLCO) compared to SSc-no ILD patients (p < 0.05; p < 0.001; p < 0.001 and p < 0.001, respectively). Of note, 30% of patients were receiving maintenance treatment with immunosuppressive drugs and 27% were receiving oral corticosteroids.

**Table 1:** Demographic and clinical characteristics of HS and SSc patients.

	HS (n = 39)	SSc-no ILD (n = 63)	SSc-ILD (n = 39)
Age, yrs	59 ± 10	55 ± 12	61 ± 12
Gender (M/F)	13/26	17/46	8/31
BMI, kg/m^2^	26 ± 4	25 ± 4	25 ± 5
Smokers (NS/FS/S)	17/16/6	31/17/15	23/11/05
Paq-year	12 ± 18	10 ± 14	6 ± 12
Haemoglobin	–	13.93 ± 1.98	12.98 ± 2.94
FEV1 post-BD, %pred	106 ± 18	99 ± 21°	88 ± 22°*
FVC post-BD, %pred	112 ± 18	105 ± 19	90 ± 22***
FEV1/FVC post-BD, %pred	78 ± 6	78 ± 10	81 ± 8
TLC, %pred	–	100 ± 15	85 ± 18***
DLCO, %pred	–	72 ± 20	57 ± 17***
KCO, %pred	-	79 ± 19	76 ± 16
Immunosuppressor (yes/no)	–	16/47	15/24
OCS (yes/no)	–	19/44	9/30
PAH/asthma (%)		4.8/0	13/2.5
UIP/NSIP/mixed pattern			2.5/10/7.5
Disease duration (y)	–	7.2 ± 8.5	6.3 ± 7.0
Rodnan skin score	–	3.4 ± 5.1	4.8 ± 6.9
ACR/Eular score	–	10.5 ± 5.6	13.7 ± 9.45
Limited SSc/lcSSc/dSSc/ SS	–	20/34/4/2	11/17/6/1
Musculoskeletal involvement (%)	–	9	16
Renal crisis (%)	–	4	0
Cardiac involvement (%)	–	2	4
GI involvement (%)	–	70	82

Data are expressed as mean ± SD. *dSSc* diffuse cutaneous SSc, *DLCO* diffusion lung capacity for CO, *FEV1* forced expired volume in 1 s, *FS* former smoker, *FVC* forced vital capacity, *GI* gastrointestinal, *HS* healthy subjects, *ILD* interstitial lung disease, *IT* immunosuppressive therapy (Mycophenolate Mofetil, methotrexate, cyclophosphamide), *KCO* the carbon monoxide transfer coefficient, *lcSSc* limited cutaneous SSc, *NS* non smoker, *NSIP* nonspecific interstitial pneumonia, *OCS* oral corticosteroid, *PAH* pulmonary arterial hypertension, *S* smoker, *SS* sine scleroderma, *SSc* systemic sclerosis, *TLC* total lung capacity, *UIP* usual interstitial pneumonia.

°*p* < 0.05 compared to HS.

*p < 0.05 and ***p < 0.001 compared to SSc-no ILD.

### Serum biomarkers at baseline

**Figure 2:**
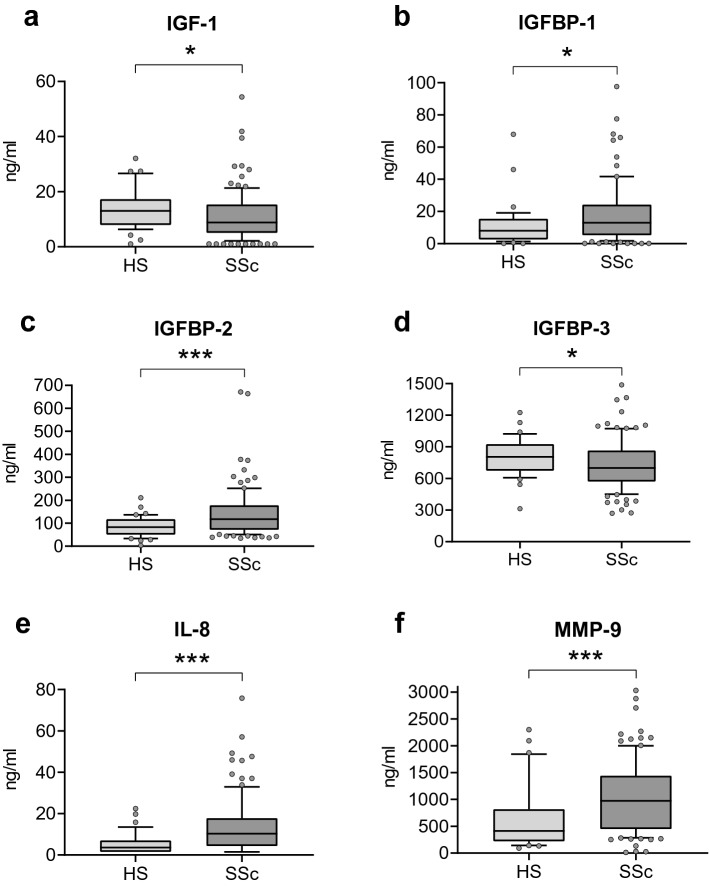
Serum biomarkers in SSc patients compared to HS. Comparison of the concentration of (**a**) IGF-1, (**b**) IGFBP-1, (**c**) IGFBP-2, (**d**) IGFBP-3, (**e**) IL-8 and (**f**) MMP-9 in SSc patients and HS. Data are expressed as median (IQR—CI 90%). *p < 0.05, **p < 0.01, ***p < 0.001 compared to HS. *HS* healthy subjects, *IGF-1* insulin like growth factor-1, *IGFBP-1*,* -2*, *-3* insulin like growth factor binding protein-1, -2, -3, *IL-8* interleukin-8, *MMP-9* matrix metalloproteinase-9, *SSc* systemic sclerosis.

First, we compared the levels of different serum biomarkers associated with lung fibrosis (total IGF-1, IGFBP-1, IGFBP-2, IGFBP-3, TGF-β1, YKL-40, and CRP)^20,40–42^, inflammatory (IL-8 and TNF-α)^43,44^ and tissue remodeling processes (MMP-7 and MMP-9)^45,46^ between HS and SSc groups (Fig. 2). There is a significant increase in IGFBP-1 (8–12.9 ng/ml, p < 0.05), IGFBP-2 (83–117 ng/ml, p < 0.001), IL-8 (3.6–9.3 pg/ml, p < 0.001), MMP-9 (412–967 ng/ml, p < 0.001) and CRP (0.7–2.1 mg/l, p < 0.001) levels in SSc patients compared to HS (Fig. 2b,c,e,f and see Supplementary Table S1). Of note, total IGF-1 and IGFBP-3 were significantly reduced SSc patients compared to HS (13–8.9 ng/ml, p < 0.05; and 806–694 ng/ml, p < 0.05, respectively) (Fig. 2a,d).

Then, we compared the levels of serum biomarkers between the two subgroups of SSc patients (SSc-ILD vs SSc-no ILD) and HS (Table 2). The level of IGFBP-2 was increased and IGFBP-3 reduced in SSc-no ILD (p < 0.05 and p < 0.001, respectively) and SSc-ILD patients (p < 0.001 and p < 0.05, respectively) compared to HS. Of note, the level of IGFBP-1 was increased only in SSc-no ILD patients compared to HS (p < 0.05). On the other side, the level of CRP was increased and total IGF-1 reduced in SSc-ILD patients compared to HS (p < 0.001 and p < 0.05, respectively). Then, we focused our analysis on the difference between patients with SSc-no ILD and SSc-ILD. Interestingly, we observed a significant reduction of the levels of IGFBP-1 and IGFBP-3 in SSc-ILD compared to SSc-no ILD patients (p < 0.01 and p < 0.05, respectively).

**Table 2:** Concentrations of serum biomarkers in subgroups of SSc patients (SSc-no ILD and SSc-ILD) and HS.

	HS (n = 39)	SSc-no ILD (n = 63)	SSc-ILD (n = 39)
IGF-1 (ng/ml)	13 (8–17)	8.9 (5.3–15.3)	9 (5.2–15.8)°
IGFBP-1 (ng/ml)	8 (3–16)	15 (7–25)°	5.8 (2.2–15.8)**
IGFBP-2 (ng/ml)	83 (51–109)	113 (69–145)°	132 (85–213)°°°
IGFBP-3 (ng/ml)	806 (675–926)	740 (598–877)°°°	656 (479–758)°*
Ratio IGF-1/IGFBP-1	5 (3–15)	2.2 (0.7–6.2)°°°	5.3 (1.4–20.6)**
Ratio IGF-1/IGFBP-2	0.7 (0.4–1.4)	0.4 (0.2–0.8)°	0.3 (0.2–0.9)°°°
Ratio IGF-1/IGFBP-3	0.1 (0–0.1)	0.05 (0.03–0.09)	0.07 (0.04–0.1)
TGF-β1 (ng/ml)	26 (24–31)	29 (24–34)	29 (22–35)
IL-8 (pg/ml)	3.6 (1.5–7)	9.3 (3.8–17.4)°°°	11 (6–19)°°°
TNF (pg/ml)	1.5 (1.5–1.5)	1.5 (1.5–1.5)	1.5 (1.5–1.5)
YKL40 (ng/ml)	33 (24–49)	37 (19–60)	46 (22–64)
MMP-7 (ng/ml)	1.7 (1.4–2)	1.7 (1–2.9)	2.4 (1.4–3.9)
MMP9 (ng/ml)	412 (221–818)	796 (413–1292)°	1183 (482–1575)°°°
CRP (mg/l)	1.2 ± 1.4	3.5 ± 4	6.4 ± 9.1°°°

Data are expressed as median (interquartile range). *CRP* C-reactive protein, *HS* healthy subjects, *IGF-1* insulin like growth factor-1, *IGFBP-1, -2, -3* insulin-like growth factor -1, -2, -3, *IL-8* interleukin-8, *MMP-7, -9* metalloproteinase-7 and -9, *SSc* systemic sclerosis, *TGF-β1* transforming growth factor-β, *TNF-α* tumor necrosing factor-α, *YKL-40* chitinase-3-like protein 1.

°*p* < 0.05 and °°°*p* < 0.001 compared to HS.

*p < 0.05 and **p < 0.01 compared to SSc-no ILD.

We also performed the molar ratio of total IGF-1/IGFBPs known as reflecting the real IGF activity. Serum molar ratio of total IGF-1/IGFBP-1 was significantly lower in SSc-no ILD patients compared to HS (p < 0.001), and total IGF-1/IGFBP-2 was significantly lower in SSc-no ILD and SSc-ILD patients compared to HS (p < 0.05 and p < 0.001, respectively). Interesting, serum molar ratio of total IGF-1/IGFBP-1 was significantly higher in SSc-ILD compared to SSc-no ILD patients (p < 0.01), suggesting an elevated level of free IGF-1 in patients with SSc-ILD.

There was a significant increase of the levels of IL-8 and MMP-9 in patients with SSc-no ILD (p < 0.001 and p < 0.05, respectively) and SSc-ILD (p < 0.001 and p < 0.001, respectively) compared to HS.

We did not find any significant relation between biomarkers and therapies at baseline (immunosuppressive agent or systemic corticosteroids).

### Correlation between serum biomarkers and pulmonary function tests at baseline

**Figure 3:**
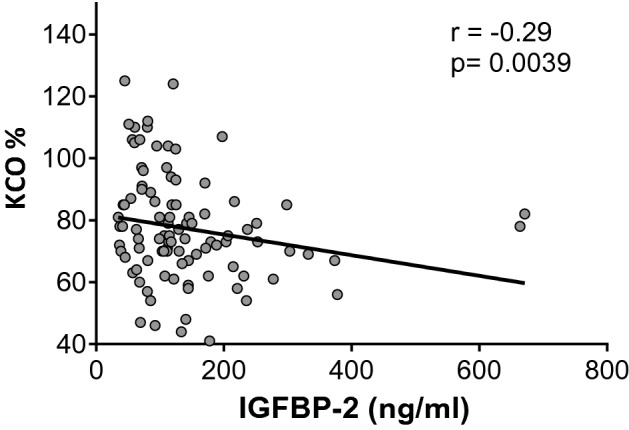
Correlation at baseline between KCO (% pred) and IGFBP-2 levels in SSc cohort. *IGFBP-2* insulin-like growth factor-2, *KCO* the carbon monoxide transfer coefficient.

We performed correlation analysis to assess whether biomarkers were associated with pulmonary function tests (PFTs) at baseline in SSc patients. IGFBP-2 was negatively correlated with alveolo-capillar function assessed by carbon monoxide transfer coefficient (KCO) (%pred) (r = − 0.29, p < 0.01) (Fig. 3). In addition, there was an inverse relationship between spirometric values and YKL-40 (FEV1%pred r = − 0.3, p < 0.01; FVC %pred r = − 0.31, p < 0.01 and DLCO %pred r = − 0.24, p < 0.05), CRP (FEV1%pred r = − 0.31, p < 0.01; FVC %pred r = − 0.32, p < 0.01 and TLC %pred r = − 0.26, p < 0.05) and total IGF-1 (TLC %pred r = − 0.23, p < 0.05) (Table 3). Interestingly, IGFBP-1 was positively correlated with TLC (%pred) (r = 0.28, p < 0.01) (Table 3).

**Table 3:** Spearman correlation evaluating serum biomarkers at baseline in comparison with pulmonary function tests.

	FEV1 %pred	FVC %pred	TLC %pred	DLCO %pred	KCO %pred
IGF-1	− 0.08	− 0.15	− 0.23*	− 0.09	0.02
IGFBP-1	0.05	0.1	0.28**	0.05	− 0.2
IGFBP-2	− 0.13	− 0.12	0.16	− 0.16	− 0.29**
IGFBP-3	0.08	0.06	− 0.01	0.07	0.12
TGF-β1	0.04	− 0.03	− 0.02	0.15	0.03
IL-8	− 0.12	− 0.06	− 0.03	− 0.12	− 0.17
TNF-α	− 0.12	− 0.07	0.14	− 0.08	− 0.15
YKL-40	− 0.3**	− 0.31**	− 0.14	− 0.24*	− 0.12
MMP-7	− 0.16	− 0.18	− 0.2*	− 0.16	− 0.05
MMP9	− 0.11	− 0.14	− 0.18	− 0.04	0.07
CRP	− 0.31**	− 0.32**	− 0.26*	− 0.13	0.15

Numbers represent the correlation coefficient (r), *p < 0.05, **p < 0.01.

*CRP *C-reactive protein, *DLCO *diffusion lung capacity for CO, *FEV1 *forced expired volume in 1 s, *FVC *forced vital capacity, *IGF-1 *insulin like growth factor-1, *IGFBP-1, -2, -3 *insulin-like growth factor -1, -2, -3, *IL-8 *interleukin-8, *KCO *the carbon monoxide transfer coefficient, *MMP-7, -9 *metalloproteinase -7 and -9, *TGF-β1 *transforming growth factor β, *TLC *total lung capacity, *TNF-α *tumor necrosing factor α, *YKL-40 *chitinase-3-like protein 1.

### Longitudinal analysis on serum biomarker variations and pulmonary function tests

To assess whether the variation over the time of the levels of serum biomarkers was associated with pulmonary function declines, we performed a longitudinal study in a subgroup of 28 SSc patients analyzing biomarkers variations and PFTs over a period of 2 years (Fig. 1). Demographic and biological characteristics of SSc patients at baseline and after 2 years are given in Table 4.

**Table 4:** Demographic and biological characteristics of SSc patients at baseline and 2-year follow-up.

	Baseline SSc (n = 28)	2-Year follow-up SSc (n = 28)
Age, yrs	57 ± 12	59 ± 12
Gender (M/F)	22/6	22/6
BMI, kg/m^2^	24 ± 4	24 ± 4
Smokers (NS/ES/S)	14/10/5	14/10/5
Paq-year	0 (0–26)	1 (0–26)
FEV1 post-BD, %pred	100 ± 20	98 ± 19
FVC post-BD, %pred	103 ± 20	101 ± 18
TLC %pred	95 ± 16	94 ± 13
DLCO %pred	66 ± 17	65 ± 13
KCO %pred	81 ± 14	73 ± 12***
ILD yes/no	8/20	11/17
IT yes/no	10/18	10/18
OCS yes/no	5/23	5/23

Data are expressed as mean (SD). *DLCO* diffusion lung capacity for CO, *FEV1 *forced expired volume in 1 s, *FS* former smoker, *FVC* forced vital capacity, *ILD* interstitial lung disease, *IT* immunosuppressive therapy (Mycophenolate Mofetil, methotrexate, cyclophosphamide), *KCO* the carbon monoxide transfer coefficient, *NS* non smoker, *OCS* oral corticosteroid, *S* smoker, *SSc* systemic sclerosis, *TLC* total lung capacity.

***p < 0.001 compared to Baseline.

**Figure 4:**
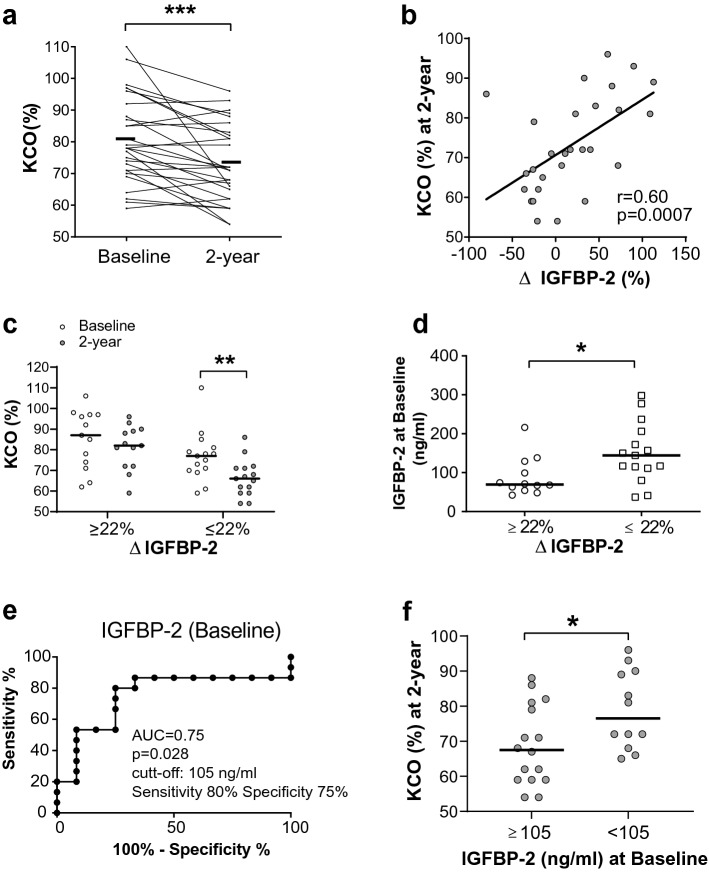
Identification of prognostic value of IGFBP-2 for the development of SSc-ILD. (**a**) KCO (%) variation in the 2-year longitudinal analysis. (**b**) Correlation study between the variation of IGFBP-2 (between baseline and 2-year follow-up) and KCO (at 2-year follow-up). (**c**) Longitudinal analysis of KCO (%) in SSc subgroups with higher or lower variation of IGFBP-2 (ΔIGFBP-2 ≥ or ≤ 22%). (**d**) Levels of baseline IGFBP-2 for SSc subgroups with higher or lower variation of IGFBP-2 (≥ or ≤ 22%). (**e**) ROC curve analysis to determine the level of baseline IGFBP-2 which will enable to discriminates the two group of SSc patients (ΔIGFBP-2 ≥ and ≤ 22%). (**f**) KCO (%) at 2-year follow-up of SSc patients with presenting baseline IGFBP-2 higher or lower than 105 ng/ml. *p < 0.05, **p < 0.01, ***p < 0.001. *AUC* area under the curve, *IGFBP-2* insulin-like growth factor-2, *KCO* the carbon monoxide transfer coefficient.

The 2-year longitudinal analysis of pulmonary function revealed that KCO was significantly reduced (Baseline: 81 (± 14) % and 2-year 73 (± 12) %, p < 0.001) (Fig. 4a). Next, we performed analysis to determine if pulmonary function decline was associated to the variation of serum biomarkers (Supplementary Table S2). Interestingly, we found a positive correlation between the variation of IGFBP-2 and KCO at 2-year follow-up (r = 0.6, p < 0.001) (Fig. 4b and Supplementary Table S2). We didn’t found any correlation between the variations of other serum biomarkers (YKL-40, CRP, IGF-1 and IGFBP-1) and PFTs (Supplementary Table S2).

Then, we investigated if IGFBP-2 could predict the progression of SSc disease. First, SSc patients were divided into two groups: patients with higher or lower variation of IGFBP-2 (∆IGFBP-2 ≥ or ≤ 22%). Interestingly, SSc patients with a lower variation of IGFBP-2 (less than 22%) presented significant deterioration of pulmonary function at 2-year follow-up (KCO %pred at baseline: 77 (± 12) % and 2-year follow-up: 66 (± 9) %, p < 0.01), whereas the ones with higher variation of IGFBP-2 (more than 22%) conserved their pulmonary function (Fig. 4c). Furthermore, baseline level of IGFBP-2 was elevated in the subgroup of SSc patients with lower variation of IGFBP-2 (less than 22%) compared the ones with higher variation of IGFBP-2 (more than 22%) (Fig. 4d). ROC curve analysis enabled us to identify that baseline IGFBP-2 of 105 ng/ml discriminate the two subgroup of SSc patients (AUC = 0.75 at 80% sensibility and 75% specificity, p = 0.028) (Fig. 4e). Indeed, baseline IGFBP-2 ≥ 105 ng/ml was associated with a poor patient’s outcome at 2-year follow-up (KCO < 70% predicted) (Fig. 4f). These results suggest that serum level of IGFBP-2 (105 ng/ml) might predict the evolution of SSc disease.

## Discussion

SSc is a complex multi-organ disorder with heterogeneous clinical features. As the diagnosis of SSc-ILD is complex, there is a need to develop novel biomarkers to identify early patients in order to deliver more appropriate treatment. Here, we quantified serum levels of several biomarkers associated with lung fibrosis, inflammatory and tissue remodeling processes in SSc patients compared to HS. SSc patients featured a marked increase in serum levels of IGFBP-1, IGFBP-2, IL-8, MMP-9 and CRP whereas total IGF-1 and IGFBP-3 were significantly reduced compared to HS. Of interest, IGFBP-2 was negatively correlated to KCO at baseline. Two-year longitudinal analysis determined that IGFBP-2 variation was positively correlated with the KCO measurement. Of great interest, initial levels of IGFBP-2 above 105 ng/ml were associated with a poor patient’s outcome 2 years later (KCO < 70% predicted), suggesting that serum levels of IGFBP-2 might predict the evolution of SSc-ILD.

In previous studies, we identified that IGFBP-2 was positively associated with lung fibrosis in serum and induced sputum of IPF patients^33,40^. Moreover, IGFBP-2 was reduced in IPF patients receiving anti-fibrotic therapy, although serum levels remained higher in IPF patients than in HS^33^. Other studies on lung fibrosis identified a significant increase of IGFBP-2 in BAL fluid and in lung tissue of ILDs without focusing on SSc^39^. In this study, we showed that patients suffering from SSc exhibited higher levels of IGFBP-2 than HS, but to a lesser extent than patients suffering from IPF (as previously shown in one of our study^33^). Of interest, we demonstrated that level variation of IGFBP-2 was associated with the severity of lung dysfunction. Indeed, baseline serum level of IGFBP-2 above 105 ng/ml allows identifying patients with a poor prognosis at 2-year follow-up (KCO < 70% predicted). This interesting observation suggests the potential prognostic value of baseline IGFBP-2 to identify SSc patients with risk of rapid evolution. Integrating new biomarkers in the follow up of SSc-ILD is challenging taking into account the variability of other clinical markers like symptoms, CRP, DLCO or FVC. Moreover, it is suitable to avoid repeated chest imaging in the follow-up of the patients to limit as much as possible irradiation. The use of serum biomarker IGFBP-2 could be a good candidate to predict the progression of SSc-ILD and need to be explored.

In our study, serum levels of TGF-β1 were similar for all groups even though TGF-β is widely known to be associated with the pathophysiology of fibrosing lung disease^47^. In a recent study, Van Caam et al. have shown that total TGF-β serum levels are not different between SSc patients and controls, but TGF-β activity is^48^. In our study, we measured levels of total TGF-β1 (not the active form); this could explain why the levels of TGF-β1 are not different between SSc patients and HS. Similarly, we did not find any difference in TGF-β levels between HS and IPF patients our previous studies^20^. In conclusion, these findings highlight that serum TGF-β is not a good biomarker of lung fibrosis.

YKL-40 was negatively correlated with PFTs (FEV1, FVC, DLCO). In accordance with previous studies^49–51^, we identified that YKL-40 was associated with the lung function impairment of patients suffering from SSc. Therefore, these observations need further explorations to see whether YKL-40 could act as a predictor of lung deterioration for SSc patients.

IL-8 was also increased in our study in SSc patients. IL-8 is known to be a strong chemotactic agent for neutrophils and can impact the pathophysiological processes of SSc by recruiting neutrophils in lungs^52,53^. Of interest, it should note that blood neutrophils were increased in SSc patients compared to HS. Furthermore, several studies have shown that SSc patients have elevated levels of pro-inflammatory cytokines such as interleukin IL-8, IL-6, TNF-α in BAL and serum^6,54,55^. In the same line, MMP-9 was also increased in SSc context. MMP-9 is known to be actively secreted by neutrophils^56,57^, which are increased in SSc patients.

Among all the molecules that we studied, only serum level of IGFBP-2 was able to predict the occurrence of ILD in SSc patients. Indeed, serum IGFBP-2 above 105 ng/ml might be a prognostic factor of alveolo-capillary dysfunction. We need to validate those results in a larger longitudinal trial to confirm the clinical value of these observations.

## Methods

### Subject characteristics

In this study, we prospectively recruited patients with SSc (SSc-ILD and SSc-no ILD) and healthy subjects (HS) from our ambulatory care policlinic at CHU Liege. The blood of the patients was collected at time of diagnosis of SSc in our center. The diagnosis of SSc was made according to the international recommendations of ACR/Eular^3^. SSc is characterized by fibrosis of the skin and visceral organs (heart, kidneys, lungs and gastrointestinal tract), narrowing of vascular lumen by intimal fibrosis leading to distal ischemia (almost constant Raynaud's phenomenon). Standard assessment exams include respectively: Rodnan score, cardiac ultrasound, urine sediment and renal biopsy, lung CT scan and PFT, esogastroduodenal transit and capillaroscopy. There are 4 forms of SSc which are distinguished by the presence of skin injury or not^58,59^. SSc with skin injury—diffuse cutaneous SSc (dSSc) for which involvement extends beyond the elbows and knees, affecting the proximal limbs and/or the trunk and—limited cutaneous SSc (lcSSc) for which the injury does not rise above the elbows and knees. In the other hand, SSc without skin injury—sine scleroderma (SS) characterized by visceral involvement, which is not the case with—limited SSc (no organ damage). SSc-ILD was defined by a combination of specific HRCT images of at least 10% of all parenchyma (reticulations, honey combing and/or ground glass opacities) with clinical signs (velcros or crackels) or symptoms (cough, shortness of breath) and alteration of PFTs. We excluded all other causes of ILD (such asbestosis, IPF, idiopathic non-specific interstitial pneumonia, hypersensitivity pneumonitis or toxic pneumonitis). All cases were validated after a multidisciplinary discussion in order to confirm the presence or absence of SSc-ILD. Then, we performed a longitudinal study, resampling blood 2 years after the first analysis (n = 28). HS were recruited by advertisement in our policlinic waiting room. They all denied any respiratory disease and had normal spirometric values with FEV1 > 80% predicted and FEV1/FVC ratio > 70%. The impact of maintenance of immunosuppressive drugs on cell count and biomarker levels was not relevant in our study. The protocol was approved by the ethics committee of CHU of Liège, and all subjects gave written consent before their enrollment (Belgian number: B707201422832; ref: 2014/302). All methods were performed in accordance with the relevant guidelines and regulations.

### Pulmonary function tests

All tests were performed according to the recommendations of the European Respiratory Society (ERS). The results were expressed in percent predicted. The total lung capacity (TLC) was measured by body plethysmography and expressed in percent predicted. The diffusion capacity of CO (DLCO) and the report DLCO/AV (alveolar volume) were measured by the single-breath carbon monoxide gas transfer method and expressed in percent predicted (SensorMedics2400He /CO Analyzer System, Bilthoven, Netherlands).

### Biomarkers measurements in serum

Levels of Interleukin (IL)-8, tumor necrosis factor (TNF)-α, matrix metalloproteinase (MMP)-7, Chitinase-3-like protein 1 (YKL-40), IGFBP-1 and IGFBP-3 were assessed by ELISA multiplex using Fluorokine-1. Multianalyte Profiling Kits (R&D Systems, Minneapolis, MN, USA) according to the manufacturer’s instructions. The detection limit for this assays were 3–3–200–230–170–705 pg/ml respectively. The concentrations of the other proteins were measured separately by ELISA: TGF-β1, MMP-9, total IGF-1, IGFBP-2 (DuoSet kit, R&D systems). The detection limits for these kits were 7–25–25–32 pg/ml respectively. In order to dissociate IGF-1 from IGFBPs, the serum samples have been pretreated in an acidic buffer, followed by the measurement of the resulting free IGF-1. The molar rations total IGF-1/IGFBPs were performed to estimate the real IGF activity.

### Statistical analysis

Demographic and functional data were expressed as mean ± standard deviation (SD). The biomarkers levels were expressed as median (IQR). Comparisons between groups were performed by Dunn's test of multiple comparisons following a significant Kruskal–Wallis test, or by Mann–Whitney or unpaired “*t*” test (according to the distribution of the variable) for pairwise comparison. Correlations between variables were performed using Spearman’s rank correlation test. A p < 0.05 was considered as significant. Statistical analysis and graph were performed with Prism Graph Pad software^®^ v6. San Diego.

### Ethics approval and consent to participate

The study protocol was approved by the ethics committee of Hospitalo-Facultaire Universitaire de Liège (CHU Hospital of Liège, Belgian number: B707201422832; ref: 2014/302). All subjects gave written consent be for their enrollment.

## Supplementary Information

Supplementary Tables.

## Data Availability

The data underlying this article are available in the article and in its online Supplementary Information. Further inquiries will be shared on reasonable request to the corresponding author.

## Abbreviations

ANA: Antinuclear antibodies
BRDU: Bromodeoxyuridine
BAL: Bronchoalveolar lavage
CRP: C-reactive protein
DLCO: Diffusion lung capacity for CO
FEV1: Forced expired volume in 1 s
FVC: Forced vital capacity
IGF-1: Insulin like growth factor-1
IGFBP-1, -2, -3: Insulin like growth factor binding protein-1, -2, -3
IL-8: Interleukin-8
ILD: Interstitial lung disease
IPF: Idiopathic pulmonary fibrosis
IQR: Interquartile range
KCO: The carbon monoxide transfer coefficient
MMP-7, -9: Matrix metalloproteinase-7 and -9
Pro-Col I: Pro-collagen type I
SD: Standard deviation
SSc: Systemic sclerosis
SSc-ILD: Systemic sclerosis associated interstitial lung disease
TGF-β1: Transforming growth factor β1
TLC: Total lung capacity
TNF-α: Tumor necrosing factor α
YKL-40: Chitinase-3-like protein 1
