# Supplementary material for: Serum IGFBP-2 in systemic sclerosis as a prognostic factor of lung dysfunction
Source: Sci Rep. 2021 May 25;11:10882. doi: 10.1038/s41598-021-90333-0 (PMC8149825; doi:10.1038/s41598-021-90333-0)
Supplement: Supplementary file 1 — Supplementary Tables. [file 41598_2021_90333_MOESM1_ESM.pdf]

ONLINE SUPPLEMENTARY INFORMATION

Serum IGFBP-2 in Systemic Sclerosis as a Prognostic Factor of Lung Dysfunction

Julien Guiot[1,†], Makon-Sébastien Njock[1,2,†,*], Béatrice André[2], Fanny Gester[1], Monique Henket[1], Dominique de Seny[2], Catherine Moermans[1], Michel G. Malaise[2] & Renaud Louis[1]

† Both authors contributed equally to this work

[1] Laboratory of Pneumology, GIGA Research Center, University of Liège, University Hospital of Liège, Liège, Belgium. [2] Laboratory of Rheumatology, GIGA Research Center, University of Liège, University Hospital of Liège, Liège, Belgium

*** Correspondence:**
Dr Makon-Sébastien Njock
ms.njock@chuliege.be

Supplementary Tables

Supplementary Table S1. Concentrations of serum biomarkers in SSc patients and HS.

	**HS (n=39)**	**SSc (n=102)**
**IGF-1 (ng/ml)**	13 (8 -17)	8.9 (5.1 – 15.3)*
**IGFBP-1 (ng/ml)**	8 (3 -16)	12.9(5.4-24)*
**IGFBP-2 (ng/ml)**	83 (51 -109)	117(73.8-177.6)***
**IGFBP-3 (ng/ml)**	806 (675 -926)	694(571-861)*
**ratio IGF-1/IGFBP-1**	5 (3 -15)	2.8(0.9-10.2)*
**ratio IGF-1/IGFBP-2**	0.7 (0.4 -1.4)	0.3(0.2-0.9)***
**ratio IGF-1/IGFBP-3**	0.1 (0 -0.1)	0.1(0-0.1)
**TGF-β1 (ng/ml)**	26 (24 -31)	29 (24 - 35)
**IL-8 (pg/ml)**	3.6 (1.5 -7)	9.3 (3.8 – 17.4)***
**TNF (pg/ml)**	ND	ND
**YKL40 (ng/ml)**	33 (24 -49)	42 (22 - 63)
**MMP-7 (ng/ml)**	1.7 (1.4 -2)	2 (1.3 – 3.5)
**MMP9 (ng/ml)**	412 (221 -818)	967 (444 - 1422)***
**CRP (mg/l)**	0.7 (0.3 -1.2)	2.1 (0.8 – 5.4)***

Data are expressed as median (interquartile range). CRP: C-Reactive Protein; HS: healthy subjects; IGF-1: Insuline like growth factor-1; IGFBP-1,-2,-3: Insuline-like growth factor -1,-2,-3; IL-8: Interleukin-8; MMP-7,-9: Metalloproteinase-7 and -9; SSc: Systemic sclerosis; TGF-β1: Transforming growth factor-β1; TNF-α: Tumor necrosing factor-α; YKL-40: Chitinase-3-like 209 protein 1.
ND : not detectable. * p<0.05 **p<0.01 ***p<0.001 compared to HS.

Supplementary Table S2. Spearman correlation evaluating pulmonary function tests at 2-year follow-up in comparison with biomarker variation (Δ) between baseline and 2-year follow-up.

	**FEV1 %pred.**	**FVC %pred.**	**TLC %pred.**	**DLCO %pred.**	**KCO %pred**
Δ **IGF-1**	0.02	0.00	0.11	0.34	0.26
Δ **IGFBP-1**	-0.09	-0.12	-0.03	-0.08	0.03
Δ **IGFBP-2**	-0.02	-0.08	-0.28	0.29	0.60***
Δ **IGFBP-3**	0.16	0.09	0.20	0.42*	0.11
Δ **TGF-β1**	0.03	0.14	0.09	0.33	0.31
Δ **IL-8**	-0.01	-0.10	-0.29	-0.08	0.09
Δ **YKL40**	0.07	-0.04	-0.07	-0.21	-0.02
Δ **MMP7**	0.05	-0.12	-0.25	-0.08	0.12
Δ **MMP9**	0.03	0.00	0.14	-0.09	-0.22

Numbers represent the correlation coefficient (r), *p<0.05, **p<0.01, *** p<0.001.
Abbreviations: DLCO = Diffusion lung capacity for CO; FEV1 = Forced expired volume in one second; FVC = Forced Vital Capacity; IGF-1 = Insuline like growth factor-1; IGFBP-1,-2,-3 = Insuline-like growth factor -1,-2,-3; IL-8 = Interleukin-8; KCO = The carbon monoxide transfer coefficient; MMP-7,-9 = Metalloproteinase -7 and -9; SSc = Systemic sclerosis; TGF-β = Transforming growth factor β1; TLC = Total lung capacity; YKL-40 = Chitinase-3-like protein 1.
